# The current status of syphilis prevention and control in Jiangsu province, China: A cross-sectional study

**DOI:** 10.1371/journal.pone.0183409

**Published:** 2017-08-24

**Authors:** Yuan-Fang Chen, Jian-Ping Ding, Hong-Jing Yan, Jing Lu, Ping Ding, Guo-Hong Chen, Jian-Jun Li, Xi-Ping Huan, Hai-Tao Yang, Wei-Ming Tang, Geng-Feng Fu

**Affiliations:** 1 Jiangsu Provincial Center for Disease Control and Prevention, Nanjing, China; 2 Jiangsu Institute of Parasitic Diseases, Wuxi, China; 3 University of North Carolina Project-China, Guangzhou, China; China Medical University, CHINA

## Abstract

**Objective:**

To analyze the midterm evaluation data from the National Syphilis Prevention and Control Plan (2010–2020) and evaluate the current status of syphilis prevention and control in Jiangsu province, China.

**Methods:**

We collected data via (1) field surveys conducted in 2015 and (2) data recorded in existing syphilis surveillance systems. We conducted descriptive statistical analysis to evaluate the current landscape of syphilis control initiatives and their potential effect in syphilis control.

**Results:**

The incidence of all cases of syphilis decreased from 2010 (32.3 per 100,000) to 2015 (30.1 per 100,000), with an annual growth of -1.17% (*x*^2^_trend_ = -7.52, P<0.001) in Jiangsu province. The incidence of primary and secondary syphilis and congenital syphilis both decreased significantly from 2010 to 2015. The average awareness rate of syphilis knowledge among professional personnel was 95.4% (3781/3963). Rural residents had the lowest awareness rate (83.5%, 1875/2245) and commercial sex workers had the highest awareness rate (92.1%, 7804/8474) in 2015. Only 47.8% (33908/70894) of patients received provider-initiated syphilis counseling and testing (PISTC) services in sexually transmitted disease (STD) clinics, but 94.5% (87927/93020) of all syphilis patients received free testing for syphilis. Overall, 97.2% (9378/9648) of syphilis reported cases of syphilis at medical institutions were confirmed to be accurate, and 92.2% (5850/6345) of patients diagnosed with syphilis at medical institutions received treatment with penicillin.

**Conclusion:**

The syphilis incidence rate in Jiangsu has decreased in recent years, but remains at a high level. It is essential to promote PISTC services to improve knowledge of syphilis and rates of testing and treatment in Jiangsu province.

## Introduction

In 2016, the World Health Organization (WHO) estimated that there are 5.6 million new cases of syphilis infections each year, with most cases occurring in developing countries [1–2]. Jiangsu, a coastal province in eastern China, has recently experienced a rise in the prevalence of sexually transmitted infections including syphilis [3]. According to China’s information system for disease control and prevention statistics, syphilis become the third most common reported infectious diseases in Jiangsu province since 2007, after tuberculosis and hepatitis B. From 2010 to 2016, 24,000 syphilis cases were reported in the province each year. There also exist several high-risk populations: the prevalence of syphilis has been estimated to be as high as 4.88% among female sex workers [4], 20.34% among men who have sex with men(MSM) [3], and 1.58%~4.93% [5–7] among rural migrants in Jiangsu province. The syphilis epidemic in these high-risk groups also poses a serious threat to the general population [8].

In response to an initiative established by WHO, the Chinese Ministry of Health (MOH) launched the National Program for Prevention and Control of Syphilis in China in June 2010 [9–10]. This program aims to decrease primary and secondary syphilis and eliminate congenital syphilis, with a specific target of less than 15 syphilis cases per 100,000 live births by 2020 [11]. To achieve this goal, Jiangsu Provincial Center for Disease Control and Prevention has augmented the national syphilis control plan with an additional comprehensive STD control plan. Initiatives include the establishment at least one medical institution per county that provides standardized medical services for syphilis patients, as well as additional STD surveillance sites in Jiangsu province.

To evaluate the potential effect of this comprehensive plan on controlling the syphilis epidemic in Jiangsu province, the Jiangsu Provincial Commission of Health and Family Planning issued and organized a midterm evaluation of the National Syphilis Prevention and Control Plan (2010–2020) in 2016. This goal of this study is to analyze data from the midterm evaluation and summarize the current status of syphilis prevention and control in Jiangsu province.

## Methods

One hundred counties and districts of cities in thirteen cities of Jiangsu province were involved in this study. To carry out this evaluation, county/district level public health bureaus collected data through existing information systems and field surveys in 2015. In order to ensure the authenticity and accuracy of the data, county (district) self-assessment, municipal review and provincial inspection work were launched. For this study, we collected de-identified surveillance data on reported cases of syphilis in 2015 as well as de-identified survey data on population awareness of syphilis.

### Surveillance data

In this study, original data were obtained through disease-monitoring systems in Jiangsu province to calculate 9 variables, including:

1. The proportion of first-detected patients in HIV counseling testing clinics and first-methadone users in methadone maintenance treatment clinics who received free testing for syphilis:
  $$=\frac{\;Numbers\;of\;first-detected\;patients\;and\;first-\;methadone\;users\;getting\;free\;testing\;for\;syphilis}{\begin{array}{l} Total\;numbers\;of\;first-detected\;patients\;in\;HIV\;testing\;clinics\;counseling\;and\;\\ first-\;methadone\;users\;in\;methadone\;maintenance\;treatment\;clinics\;in\;2015 \end{array}};$$
2. The proportion of first-detected patients and first-methadone users with positive syphilis tests who were referred to a STD clinic for syphilis professional treatment in HIV counseling and testing clinics and methadone maintenance treatment clinics:
  $$=\frac{\begin{array}{l} \;Numbers\;of\;first-detected\;patients\;and\;first-\;methadone\;users\;with\;positive\;syphilis\\ \;having\;been\;referred\;for\;specialized\;follow-up\;treatment \end{array}}{\begin{array}{l} First-detected\;patients\;and\;first-methadone\;users\;with\;positive\;syphilis\;in\;HIV\;counseling\;\\ and\;testing\;clinics\;and\;methadone\;maintenance\;treatment\;clinics\;in\;2015 \end{array}};$$
3. The proportion of pregnant women at public medical institutions who received free syphilis testing at least once during pregnancy:
  $$=\frac{\begin{array}{l} \;Numbers\;of\;pregnant\;women\;getting\;free\;testing\;for\;syphilis\;\\ at\;least\;once\;time\;during\;pregnancy\;in\;public\;medical\;institutions \end{array}}{Pregnant\;women\;in\;2015};$$
4. The proportion of syphilis-positive pregnant women at public medical institutions who received standard diagnostic and treatment services:
  $$=\frac{\;\;Numbers\;of\;syphilis\;positive\;women\;accepting\;standard\;diagnostic\;and\;treatment\;services}{Syphilis\;positive\;pregnant\;women\;in\;public\;medical\;institutions\;in\;2015};$$
5. The proportion of live babies born to syphilis-positive mothers who received standard syphilis diagnostic and treatment services:
  $$=\frac{\;\;Numbers\;of\;babies\;accepting\;standard\;diagnostic\;and\;treatment\;services}{Alive\;babies\;born\;to\;syphilis-positive\;mothers\;in\;2015};$$
6. The proportion of syphilis-positive mothers with one-year-old babies who accepted syphilis follow-up for their child during its first year of life:
  $$=\frac{\;\;Numbers\;of\;babies\;accepting\;follow-up\;in\;the\;first\;year\;of\;life}{One-years-old\;babies\;born\;to\;syphilis-positive\;mothers\;in\;2015};$$
7. The diagnostic accuracy rate of syphilis cases reported by medical institution: we considered each syphilis case as correctly diagnosis if the patient had records of positive tests for both a treponema pallidum serum test and a non-treponema pallidum serum test:
  $$=\frac{Syphilis\;cases\;diagnosed\;accurate}{Sample\;syphilis\;cases\;reported\;by\;surveyed\;medical\;institution\;in\;2015};$$
8. The annual growth rate in incidence of all cases of syphilis from 2010 to 2015(%):
  $$=\sqrt[{6}]{\frac{\;\;The\;reported\;incidence\;rate\;of\;all\;cases\;of\;syphilis\;in\;2015}{The\;reported\;incidence\;rate\;of\;all\;cases\;of\;syphilis\;in\;2010}}\times 100-100$$
9. The compound annual growth rate in incidence of primary and secondary syphilis from 2010 to 2015(%):
  $$=\sqrt[{6}]{\frac{\;\;The\text{ compound }incidence\;rate\;of\;primary\;and\;secondary\;syphilis\;in\;2015}{The\;compound\;incidence\;rate\;of\;primary\;and\;secondary\;syphilis\;in\;2010}}\times 100-100;$$
10. The annual growth rate in incidence of congenital syphilis from 2010 to 2015(%)
  $$=\sqrt[{6}]{\frac{\;\;The\;reported\;incidence\;rate\;of\;congenital\;syphilis\;in\;2015}{The\;reported\;incidence\;rate\;of\;congenital\;syphilis\;in\;2010}}\times 100-100.$$

### Field surveys

#### Awareness of syphilis-related knowledge

The National Center for STD Control, China CDC created a questionnaire exam of 322 items on syphilis prevention, treatment and testing to evaluate the knowledge of technical staff/medical personnel (S1 Table). In each county (district), the local center for disease control and prevention (CDC), a maternity and child health care hospital, and the three medical institutions with the largest number of reported syphilis cases during 2015 were recruited. At each institution, 3 syphilis prevention and control staff, 3 syphilis testers and 3 syphilis clinicians were given the exam. For any category with fewer than 3 participants, all individuals within that group were given the exam. Participants required a score of 85% or higher to pass the exam.

The National Center for STD Control, China CDC created a survey with eight questions on knowledge of syphilis prevention intended for residents (S2 Table). This survey was conducted among urban and rural residents in each city. In order to ensure adequate representation of survey participants, the survey was administered to 200 urban residents aged 15 to 49, 150 rural residents aged 15 to 49, 100 rural-to-urban migrants over age 18, 650 commercial female sex workers over age 18, and 300 MSM over age 18. Participants who correctly answered six or more questions were considered proficient in knowledge of syphilis prevention.

#### Provider-initiated syphilis testing and counseling (PISTC) services

Three hospitals in each county with the highest number of reported syphilis cases during 2015 were included in this survey (S3 Table). To calculate this, we used the following formula designed by China CDC: PISTC proportion = the number of dermatology outpatients who were tested for syphilis in the fourth quarter / (the number of dermatology outpatients in fourth quarter×3%).

To get the denominator, the total number of dermatology outpatients from the fourth quarter of 2015 in each selected hospital was collected. The numbers of patients being tested for syphilis in the fourth quarter of 2015 at the selected hospitals were also collected and used as nominator.

#### Investigation on standard treatment of syphilis

Long-acting penicillin is widely regarded as the best treatment for syphilis [12–13], and a long-acting penicillin prescription was considered to be the standard treatment in this study.

The top three hospitals for syphilis cases in each county (district) in 2015 were included in this study (S4 Table). We randomly selected 30 syphilis treatment prescriptions from each hospital and counted the number of prescriptions for long-acting penicillin. Since some patients with syphilis are allergic to penicillin, the Chinese CDC used 0.75 as a correction factor to account for patients with penicillin allergies. The standard treatment ratio was calculated as follows:

Standard treatment ratio (%) = (long-acting penicillin prescriptions/0.75) / sample syphilis treatment prescriptions×100%.

### Data analysis

Data were analyzed using SAS 9.3. We analyzed binary data and stratified data using the *CMH-x*^2^ test (Cochran-Mantel-Haenszel chi-squared test). We also examined the trends in reported incidence rate of syphilis from 2010 to 2015. Spatial analysis was integrated using geographic information system software (GIS 10).

## Results

### Reported incidence of syphilis

**Fig 1:**
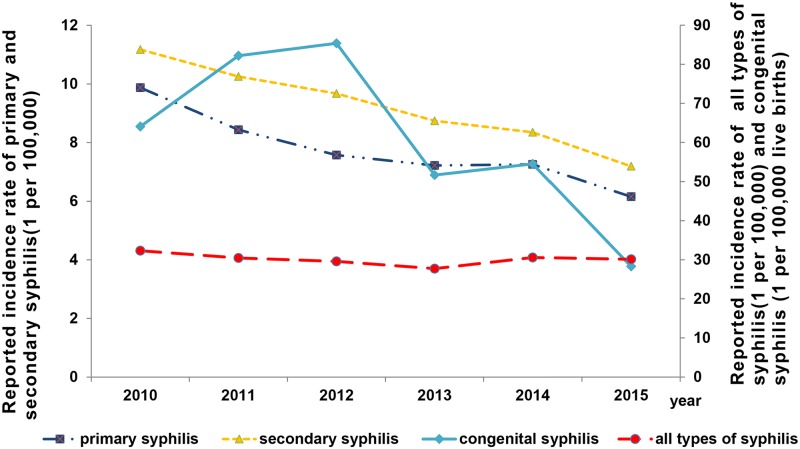
The reported incidence rate of syphilis from 2010 to 2015 in Jiangsu province. The incidence of syphilis decreased overall, and the incidence of primary and secondary syphilis and congenital syphilis both decreased significantly from 2010 to 2015.

The incidence of all cases of syphilis decreased from 2010 (32.3 per 100,000) to 2015 (30.1 per 100,000), with an annual growth of -1.17% (*x*^2^_*trend*_ = -7.52, *P*<0.001) (Fig 1). The combined incidence of primary and secondary syphilis decreased significantly from 21.0 per 100,000 (16259 cases) in 2010 to 13.3 per 100,000 (10615 cases) in 2015, with an annual growth rate of -7.32% (*x*^2^_*trend*_ = -39.90, *P*<0.001) (Fig 1). The incidence of congenital syphilis decreased significantly from 64.1 per 100,000 live births (619 cases) in 2010 to 28.3 per 100,000 live births in 2015 (224 cases), with an annual growth rate of -12.75% (*x*^2^_*trend*_ = -12.31, *P*<0.0001) (Fig 1).

### Awareness of syphilis prevention and treatment knowledge

#### Professional personnel survey

A total of 3963 professional personnel participated in the syphilis prevention, knowledge, and skills examination survey. 3781 (95.4%) of examinees passed the examination (Table 1). The awareness rate of syphilis knowledge among professional personnel was 98.5% (666/676) at the Jiangsu CDC, 94.7% (2264/2390) at the three medical institutions with most cases of syphilis, and 94.9% (851/897) at the maternity and child health care hospital. The difference among the three groups was significant (*x*^2^ = 14.45, *P*<0.001) (Table 1). The awareness rate of syphilis knowledge was 97.1% (965/994) in syphilis prevention and control staff, 95.9% (1384/1443) in syphilis testing staff, 93.8% (1432/1526) in syphilis clinicians. The difference was significant among these groups of participants (*x*^2^ = 10.56, *P* = 0.005).

**Table 1:** Awareness of syphilis prevention and treatment knowledge among professional personnel in Jiangsu, China, 2015 (n = 3963).

Participant	Total	CDC		Medical institutions		Maternity and child health care hospitals	
		Participants	Eligible rate(%)	Participants	Eligible rate(%)	Participants	Eligible rate(%)
Syphilis prevention and control staff	994	226	98.7	538	96.1	230	97.8
Syphilis testing staff	1526	128	97.7	1032	94.0	366	92.1
Syphilis clinicians	1443	322	98.8	820	94.8	301	96.0
Total	3963	676	98.5	2390	94.7	897	94.9

#### Residents’ survey

Rural residents had the lowest rate of proficiency in knowledge of syphilis at 83.5% (1875/2245), followed by rural-to-urban migrants at 88.9% (1246/1401), urban residents at 90.9% (2530/2784), MSM at 90.8% (3472/3824), and commercial sex workers at 92.1% (7804/8474). There were statistically significant differences in the awareness of syphilis among these groups of participants (*x*^2^ = 155.1, *P*<0.001).

### PISTC services offered by medical institutions

A total of 250 dermatology hospitals and STD clinics were investigated for the rate of PISTC services offered to their patients. 47.8% (33908/70894) of patients received PISTC services in the fourth quarter of 2015, which is lower than the national standard (PISTC proportion> = 80%). Of the 13 surveyed cities in the province, only 4 cities met the national assessment standard, including Taizhou (88.2%), Yangzhou (87.4%), Lianyungang (83.2%) and Huai’an (80.9%)(Table 2).

**Table 2:** The PISTC among 70894 patients in 250 medical institutions with STD clinic in Jiangsu, China, 2015.

City	PISTC proportion <80%		PISTC proportion > = 80%		Average PISTC proportion(%)
	STD outpatients	Proportion (%)	STD outpatients	Proportion(%)	
Nanjing	10944	33.9	1329	93.8	40.4
Suzhou	21095	21.3	3918	98.2	33.4
Wuxi	3036	42.1	1725	89.6	59.3
Nantong	1924	44.4	1745	94.4	68.2
Yangzhou	-	-	1311	87.4	87.4
Changzhou	3173	63.9	1120	99.0	73.1
Yancheng	591	42.1	1165	91.9	75.2
Zhenjiang	1009	59.4	1753	92.0	80.1
Xuzhou	7427	21.2	1282	92.9	31.8
Taizhou	-	-	1733	88.2	88.2
Huai-an	528	40.5	1494	95.1	80.9
Lian-yungang	102	38.2	689	89.8	83.2
Suqian	1073	24.8	728	85.0	49.1
Total	50902	30.1	19992	93.1	47.8

Of the 250 surveyed medical institutions, 94 (37.6%) did not meet the national assessment standard (e.g. the proportion of patients receiving PISTC services in these medical institution was less than 80%) (Table 2). There were a total of 50902 outpatients in those 94 institutions in the fourth quarter in 2015, and the average rate of PISTC services was only 30.1% (15305/50902). Each medical institution had an average of 200 outpatient dermatology patients per day. In the 156 institutions (62.4%) that met the national assessment standard (PISTC proportion> = 80%), 19992 people were seen for STD outpatient care during the fourth quarter in 2015, with an average PISTC coverage of 93.0% (18603/19992).

### Syphilis monitoring and detection among high-risk populations

**Fig 2:**
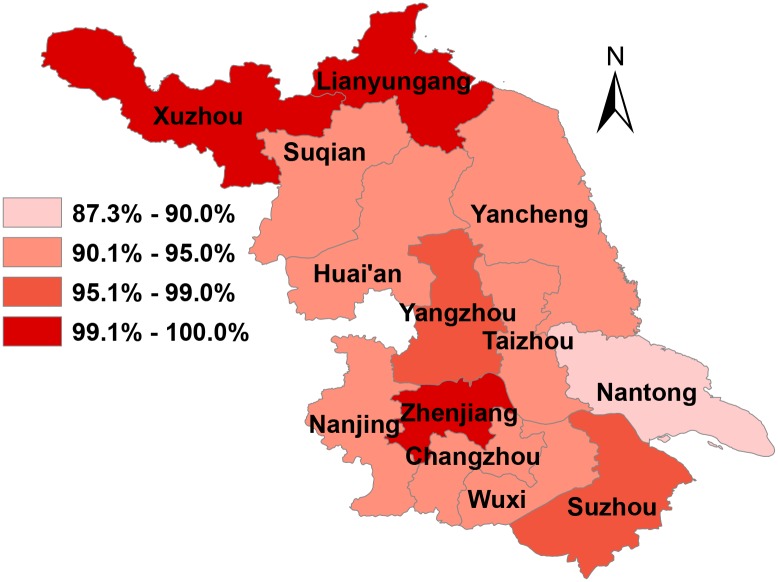
The proportion of first-detected patients at HIV clinics and first-methadone users at methadone clinics who received free testing for syphilis in 2015. Zhenjiang, Lianyungang and Xuzhou had the highest proportion (>99%), while Nantong had the lowest proportion (87.3%).

As of 2015, there are 356 HIV counseling and testing clinics and 17 methadone maintenance treatment clinics in Jiangsu province. The proportion of first-detected patients at HIV counseling and testing clinics s and first-methadone users at methadone clinics who received free testing for syphilis was 94.5% (87927/93020) throughout the province and rate of testing in each city was above 85% (Fig 2). The percentage of patients who firstly received syphilis testing at HIV clinics was 94.5% (87624/92710) and the percentage of first-methadone users who received syphilis testing at methadone maintenance treatment clinics was 97.7% (303/310).

**Fig 3:**
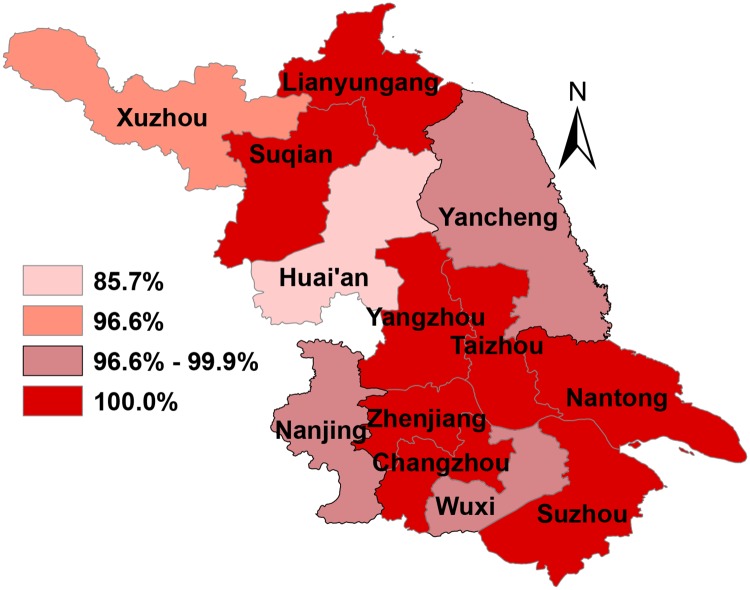
The proportion of first-detected patients at HIV clinics and first-methadone users at methadone clinics with positive syphilis tests who were referred for specialized follow-up treatment in 2015 in Jiangsu province. Lianyungang, Suqian, Yangzhou, Taizhou, Zhenjiang, Changzhou, Suzhou, and Nantong had the highest proportion (100%), while Huai’an had the lowest proportion (85.7%).

99.7% of patients with syphilis detected in these settings were referred for specialized follow-up treatment (4425/4438), and more than 85.0% of patients in each of the 13 cities received these referrals (Fig 3). The percentages of first-detected patients at HIV clinics and first-methadone users at methadone treatment clinics with positive syphilis tests who were referred to an STD clinic for syphilis follow-up were 99.7% (4336/4348) and 98.9% (89/90), respectively.

### Prevention of mother-to-child transmission (MTCT) of syphilis

In 2015, 643,452 (99.7%) urban and 152,506 (99.8%) rural pregnant women received free testing for syphilis at least once during pregnancy. The difference among the dissimilar area was significant (*x*^2^ = 1335.3, *P*<0.001) (Table 3). Of these women, 974 and 227 pregnant women with syphilis received standardized diagnostic and treatment services, representing 91.2% (974/1068) and 81.1% (227/280) of all pregnant women with syphilis, respectively. The difference among the dissimilar area was significant (*x*^2^ = 11.0, *P* = 0.004) (Table 3).

**Table 3:** Test and treatment among pregnant women and babies born to syphilis-positive mothers in urban area and rural area in Jiangsu, China 2015.

Index	South Jiangsu^a^		Center Jiangsu^b^		North Jiangsu^c^	
	Urbanareas	Rural areas	Urbanareas	Rural areas	Urban areas	Rural areas
1.Percentage of pregnant women who received a free syphilis test(n = 797712)	99.9	99.9	99.3	100.0	99.8	99.6
2.Percentage of syphilis-positive pregnant women who received standardized services (n = 1348)	89.0	76.0	96.0	87.2	91.5	82.1
3.Percentage of babies born to syphilis-positive mothers who received standard treatment services(n = 1301)	86.8	93.3	95.9	92.1	91.7	71.5

^a^: South Jiangsu includes Nanjing city, Zhenjiang city, Suzhou city, Wuxi city and Changzhou city;

^b^: Central Jiangsu includes Yangzhou city, Taizhou city and Nantong city;

^c^: North Jiangsu includes Xuzhou city, Lianyungang city, Suqian city, Huai’an city and Yancheng city.

90.0% (927/1030) of live babies born to syphilis-positive mothers in urban areas and 80.4% (218/271) of live babies born to syphilis-positive mothers in rural areas received services through standardized diagnostic and treatment programs. The difference among the dissimilar area was significant (*x*^2^ = 11.5, *P* = 0.003) (Table 3). 93.3% (938/1005) of live babies born to syphilis-positive mothers and aged over 12 month in 2015 received follow-up for syphilis during their first year of life.

### Standardized treatment and monitoring for syphilis positive patients

A total of 6345 syphilis treatment prescriptions were reviewed. 92.2% of syphilis patients received penicillin treatment. All 13 cities surveyed in Jiangsu province reached the national standard for penicillin treatment (> = 80%) in 2015.

A total of 9648 reported syphilis cases were examined in this study, and we regarded syphilis case as correctly diagnosis in condition that patient record was fully featured and patient had been tested positive for both treponema pallidum serum test and non-treponema pallidum serum test. 97.2% of reported cases from medical institutions surveyed were accurate. Accuracy rates exceeded 90% in all 13 cities.

## Discussion

This study evaluates the current status of syphilis prevention and treatment in Jiangsu province. The results suggest that Jiangsu performs well on public education, monitoring and detection of syphilis in high risk populations, prevention of mother-to-child syphilis transmission, and treatment and follow-up for syphilis patients. The incidence of syphilis decreased from 2010 (32.3 per 100,000) to 2015(30.1 per 100,000), and by 2015, the incidence of syphilis was relatively lower than Guangdong province (52.55 per 100,000 in 2014) [14] but higher than Yunnan province (27.69 per 100,000 in 2014) [15].

The proportion of patients in STD clinics who received PISTC services was only 47.8% in the fourth quarter of 2015 in Jiangsu province, which is only slightly higher than in Guangdong province (40% in 2014) [16]. Previous research has demonstrated that among outpatients at STD clinics, 19.0%~24.6% percent of patients will test positive for syphilis [17–19]. Hospital dermatology and STD clinics serve as an important line of defense for identifying patients at high risk of syphilis [20]. Medical institutions should promote PISTC services; especially they were among the institutions where PISTC services were offered to fewer than 80% of all patients. In particular, clinicians at these institutions should receive more extensive training in syphilis prevention knowledge and PISTC services. At the same time, we should improve patient education so that patients feel comfortable seeking help from healthcare providers after unsafe sex [21].

Prevention of MTCT of syphilis is a major issue worldwide [22–25]. Nearly one million pregnant women worldwide are infected with syphilis each year [26]. Antenatal syphilis screening and treatment has been shown to prevent mortality and morbidity. In 2011, the Chinese MOH launched a successful national prevention and control program to address MTCT of HIV, syphilis and hepatitis B. Afterwards, the reported incidence of congenital syphilis dropped from 79.12 cases per 100,000 live births in 2011 to 40.74 cases per 100,000 live births in 2015 [27]. The reported incidence of congenital syphilis was 28.3 cases per 100,000 live births in Jiangsu province in 2015, decreased from 64.1 cases per 100,000 in 2010, and below the national average.

In general, primary and secondary syphilis are together referred to as early cases, which are highly infective and reflect the epidemiological status of syphilis. If primary and secondary syphilis were diagnosed early and treated systematically, the transmission of syphilis could be stopped altogether [28–29]. Our study found that the reported incidence rate of primary and secondary syphilis has declined at an annual rate of 7% from 2010 to 2015 in Jiangsu province. However, this is still far higher than the incidence of primary and secondary syphilis in the United States (5.3 per 100,000 in 2013) [30]. The distribution of primary and secondary syphilis is strongly affected by many social and demographic factors, including age, education, economy, and local funding to prevent and control syphilis [31]. Further studies are needed to understand the social and behavioral factors driving the spread of syphilis in Jiangsu province [32].

This study has several limitations. First, although we can suggest some reasons for the low rates of PISTC in hospitals, we cannot draw decisive conclusions based on the evidence from our study. Further investigation is needed to determine the factors associated with low PISTC rates. Secondly, given the sheer volume of data, we only collected and analyzed surveillance data from 2015, and thus cannot offer comparisons between years to obtain information on dynamic changes. Third, the patient awareness survey of syphilis prevention and treatment did not take into account socio-demographic factors, and may be biased in favor of populations that are relatively well-informed or relatively poorly-informed. Additionally, for this survey, we did not identify populations who fell into more than one category, such as rural-to-urban migrants who were also female sex workers, even though these two populations often overlap.

## Conclusion

This study reports the current status of syphilis prevention and control in Jiangsu province, China. The incidence of syphilis has decreased in recent years, but remains at a high level. Our study can help inform current and future policies on syphilis prevention and control. It is essential to promote PISTC services and improve public knowledge of syphilis in order to control the transmission and spread of syphilis in Jiangsu province.

## Supporting information

S1 Table Test questions of syphilis prevention, diagnosis and treatment and laboratory. (DOC)

S2 Table Questionnaire about syphilis prevention knowledge. (DOC)

S3 Table Survey on provider-initiated syphilis testing and counseling (PISTC) services in STD clinics. (DOC)

S4 Table Survey on the ratio of syphilis-positive patients who had received standard treatment. (DOC)
