# Supplementary material for: The current status of syphilis prevention and control in Jiangsu province, China: A cross-sectional study
Source: PLoS One. 2017 Aug 24;12(8):e0183409. doi: 10.1371/journal.pone.0183409 (PMC5570431; doi:10.1371/journal.pone.0183409)
Supplement: S1 Table — (DOC) [file pone.0183409.s001.doc]

**S1 Table. Test questions of syphilis prevention, diagnosis and treatment and laboratory.**

| **Syphilis Prevention and Control Test Questions**  **1. Single choice**   1. According to the state regulations, which kinds system was choosing in syphilis cases report?   A. Positive reporting system B. Admissibility reporting system  C. Diagnostic reporting system D. First doctor’s reporting system   1. According to the national requirements, which choose is meet syphilis report cases? 2. Meet the national diagnostic criteria for the first case 3. Meet the national diagnostic criteria 4. Meet the Complications of national diagnostic criteria 5. Cases with clinical symptoms 6. How to fill the "infectious disease report card" when a patient is suffering from syphilis, gonorrhea and genital warts? 7. All sexually transmitted diseases only fill a card, the disease only check the syphilis 8. Syphilis fill a newspaper card, other sexually transmitted diseases to fill another card 9. Gonorrhea fill out a newspaper card, other sexually transmitted diseases to fill another card 10. Every kind of sexually transmitted diseases to fill a newspaper card 11. Syphilis case report by the first doctor is responsible for reporting, which is right? 12. By the admissions doctor to make a diagnosis after the report 13. By the first doctor to make a diagnosis of the patient report 14. Reported by the consultation doctor 15. Before the patient was diagnosed in other hospitals have been diagnosed, but in the hospital for the initial diagnosis, by the diagnosis of doctors report 16. When the doctor to fill the syphilis case "infectious disease report card" accidentally wrong, need to modify the card, which of the following is correct? 17. Report card can be modified 18. After the card is modified, it should be modified by the doctor 19. Modified by the insurance doctor, the amendment does not need to ask the card doctors 20. Report card cannot be modified 21. Which of the following is the best option for filling in syphilis case "infectious disease report card"? 22. Fill in with a pen 23. Report card should be filled correctly, complete, no missing items 24. Should be completed by the first doctor, and signed 25. Above all right 26. According to the Ministry of Health issued the diagnostic criteria, who is the right for syphilis classified? 27. Early syphilis, late syphilis 28. First syphilis, Second syphilis, Third syphilis, Recessive syphilis, Fetal syphilis 29. Dominant syphilis, recessive syphilis 30. Acquired syphilis, congenital syphilis 31. Which of the following is correct for the time limit for syphilis case reports? 32. syphilis cases should be reported within 24 hours after diagnosis 33. Syphilis cases should be reported after 3 working days 34. Syphilis cases detected positive, should be reported within 24 hours 35. Syphilis cases positive, should be reported in 3 working days 36. Which of the following is correct about the reporting requirements for syphilis referral cases? 37. Reported by the original referral doctor 38. By the referral doctor in a clear case for the first reported after the report 39. The original referral doctor and the referral doctor should report 40. No report 41. Which of the following is correct about the reporting requirements for syphilis consultation cases? 42. Consultation for the first case after the clear, by the original admissions doctor report 43. consultation for the first case after the diagnosis, by the consultation doctor report 44. original admissions doctors and consultation doctors have to report 45. No report 46. According to the Ministry of Health issued the diagnostic criteria, who is the right for Syphilis diagnosis case classification? 47. Confirmed cases, clinical diagnosis of cases 48. Confirmed cases, suspected cases 49. Clinical diagnosis of cases, pathogen carriers 50. Confirmed cases, positive test 51. Which is right contains in the first time syphilis confirmed cases? 52. Polymorphic rash, RPR and TPPA were positive 53. Hard chancre, RPR and TPPA were positive 54. Hard chancre, RPR positive 55. Chancre, TPPA positive 56. Which is right contains in the Second time syphilis confirmed cases? 57. Polymorphic rash, RPR and TPPA were positive 58. Hard chancre, RPR and TPPA were positive 59. Hard chancre, RPR positive 60. Chancre, TPPA positive 61. Which are right contains in Recessive syphilis confirmed cases？ 62. Treatment asymptomatic, no signs, previously not diagnosed with syphilis, RPR and TPPA were positive 63. Treatment asymptomatic, no signs, had previously diagnosed with syphilis, RPR and TPPA were positive 64. Treatment asymptomatic, no signs, RPR positive 65. Treatment asymptomatic, no signs, TPPA positive 66. A pregnant woman who was hospitalized in your hospital was tested for two types of syphilis serum antibody (TP-ELISA and RPR). The maternal had a history of syphilis a year ago. Does the case need to be reported?   A. Need to report B. No need to report  C. Report after Follow-up D. Do not know   1. A hospital on an inpatient ELISA screening, the results of ELISA positive, and then further RPR detection, RPR also positive, and the titer of 1: 4. The patient had no history of syphilis. Does the case need to be reported?   A. Need to report B. No need to report  C. Report after Follow-up D. Do not know   1. A local disease prevention and control center AIDS counseling and detection clinic for a doctor at the same time to carry out HIV and syphilis serum test, the results of ELISA and TRUST were positive. The treatment of this case is: 2. Immediately fill out the report on infectious disease report card 3. Not reported, referral to STD clinic, the latter to determine whether to report 4. Do not report, nor referral 5. Do not know 6. When the blood donors was found that they are syphilis seropositive by blood banks, how to deal with them? 7. Not reported, nor referral 8. Reported, Referral 9. Not reported,Referral to STD clinic 10. Do not know 11. How to deal with syphilis suspected cases of the second report of the network reported? 12. Fix the diagnosis as soon as possible and delete the case if it can not be corrected 13. The case was removed directly on the network on December 31 of this year 14. If it cannot be corrected, keep the case on the network and do not delete it 15. Do not know 16. The diagnosis of syphilis is very complicated. If the patient can not be diagnosed immediately by the existing laboratory test results, the diagnosis should be carried out by follow-up test. For the children with positive TPPA test, the final time of follow-up is:   A. 3 months B. 6 months  C. 12 months D. 18 months   1. Some people without syphilis clinical manifestations, past no syphilis diagnosis, the test results for the RPR-positive, TPPA negative, the appropriate diagnosis is:   A. First syphilis B. Second syphilis  C. Recessive syphilis D. Not syphilis   1. A senior doctor in the work of the diagnosis of a case of hidden syphilis, which of the following is correct: 2. Reported by an intern 3. Reported by a training doctor 4. Reported by graduate students 5. reported By himself 6. A patient in A city hospital diagnosis of recessive syphilis (a hospital has been reported cases), the patient is not assured, but also to the B City Hospital, hospital doctors by asking history, that the patient in the city of A hospital has been Diagnosed as recessive syphilis, after examination and testing, is still diagnosed as recessive syphilis, which of the following is correct: 7. The second hospital does not need to report 8. The second hospital needs to report 9. Report after Follow-up 10. Do not know 11. About the outpatient log of the doctor diagnose the syphilis case, which of the following is the appropriate option? 12. Only registered as syphilis, no stage 13. Detailed registration of syphilis staging 14. Do not registration 15. Do not know 16. Which of the following is a suitable option when the inpatient doctor completes the syphilis diagnosis name for hospitalized medical records? 17. Only diagnosed and filled as syphilis, not in stages 18. Diagnosis and registration of syphilis cases 19. Do not registration 20. Do not know 21. A baby to the hospital, the father said his mother have syphilis, the specific situation doctors cannot know that the baby test results for the RPR negative, TPPA positive. Which of the following is the appropriate option? 22. Immediately reported fetal transmission syphilis 23. According to follow-up results to determine whether to report 24. Do not registration 25. Do not know 26. The baby's mother had syphilis during pregnancy, the hospital for syphilis detection of the baby, the test results for the RPR titer of 1: 8, TPPA positive, the baby's mother in the test when the syphilis test results for the RPR Titer 1: 1, TPPA positive. Which of the following is the appropriate option?   A. Immediately reported fetal transmission syphilis  B. According to follow-up results to determine whether to report  C. Do not registration  D. Do not know   1. A patient go to the hospital STD clinic, the doctor asked the history of the genital area had ulcers 4 months ago, and now there is no symptoms. the test results for the RPR positive, TPPA positive, the doctor should be diagnosed as:   A. First syphilis B. Second syphilis  C. Recessive syphilis D. Not syphilis   1. A hospital for the convenience of infectious diseases report, the electronic "infectious disease report card" to join the hospital information system, but the electronic version of "infectious disease report card" syphilis diagnosis options for "syphilis"( not staging). When the doctor diagnoses the second syphilis, which of the following is the appropriate option? 2. Continue to fill out the electronic card, select "syphilis" 3. Fill in the paper card, do not fill out the electronic card 4. Do not fill any card 5. Do not know 6. A hospital has been established an infectious and operating disease network report account. According to the admissions of the first case of syphilis, which of the following options for the appropriate? 7. Do not registration 8. Fill in the report of infectious diseases card to report to the sub-disease control center 9. To avoid being punished, and diagnosed as eczema 10. Do not know 11. A hospital for the convenience of infectious diseases report, the electronic "infectious disease report card" to join the hospital information system, but the electronic version of "infectious disease report card" syphilis diagnosis options for "syphilis"( not staging). When the doctor diagnoses the second syphilis, which of the following is the appropriate option? 12. Enter a random selection of a syphilis staging 13. Immediately contact with the card reader, check syphilis and then enter the staging 14. fill Select first time syphilis 15. Do not know 16. A local disease prevention and control center staff to a hospital for data quality check and found that this year there are 5 cases of syphilis case diagnosis staging error, how to deal with? 17. Remove the case and report it again 18. Revised in the Infectious Diseases Information System and filled the revised report card 19. Do not deal with 20. Do not know 21. A pregnant woman has syphilis, and did not receive timely treatment, the hospital neonatal syphilis serum detection for baby. the results of RPR and TPPA were negative. Which of the following is appropriate for the case report on newborns? 22. Due to neonatal RPR and TPPA were negative, indicating that the newborn did not suffer from syphilis, do not report fetal syphilis, not follow-up 23. Do not report fetal syphilis, but need to follow up, according to follow-up results to determine whether the need to report 24. Due to the mothers are not timely treatment of syphilis, newborns should be immediately reported as fetal syphilis 25. Do not know 26. Which year was China's prevention and control syphilis plan?   A. 2000 B. 2005 C. 2010 D. 2015   1. In addition to AIDS, the current focus on prevention and control of sexually transmitted diseases in China are:   A. 3 B. 2 C. 5 D. 32   1. Syphilis effective personal precautions methods are: 2. Eat administration or antibiotics before and after Sex 3. Use condoms correctly during sex 4. Using soap and water to clean the genitals Before and after sex 5. Using effective sterilization drugs after sex 6. Which sexual diseases can transmit with placenta infection?   A. Human papillomavirus B. Gonorrhoeae  C. Treponema pallidum D. Haemophilus multiflora   1. Which of the following measures is the first precautionary measure for syphilis: 2. Using condoms correctly 3. Screening for sexually transmitted diseases 4. Use effective therapeutic drugs 5. Screening for positive recipients 6. By the end of 2015, China's prevention and control of syphilis plan in the national one and two syphilis report incidence rate of growth control: the following population is the focus of syphilis screening, except:   A. Sex workers B. VCT outpatient clinic  C. College Students D. Sexually transmitted disease   1. By the end of 2015, China's prevention and control of syphilis in the plan proposed that the national first and second stage of syphilis report rate of increase in the rate of control less than?   A .3% B .5% C. 8% D 10%   1. What is the type of STD / AIDS prevention measures that encourage young people to avoid premarital sex?   A. Level 1 prevention B. Level 2 prevention  C. Level 3 prevention D. none of above   1. Which of the following acts is more dangerous for sexually transmitted diseases? 2. There is no protective oral sex between men 3. sex with sex workers do not use condom 4. sex with net-friends 5. sex with sex workers have non-inserted sexual behavior 6. Which of the following is an important indicator of the evaluation of sexually transmitted diseases?   A. Change with sexual concept B. Condom use rate  C. The prevalence of STD among high risk populations  D. The awareness rate of STD prevention knowledge in different people  2. **Judgment questions**   1. According to state regulations, syphilis cases report implement of positive testing system. 2. According to state regulations, syphilis cases report implement admissibility reporting system. 3. According to the national requirements, syphilis reported cases should have clinical symptoms of the case. 4. According to national requirements, syphilis report cases should meet the national diagnostic criteria of the first case 5. According to state regulations, syphilis cases and reports implement the first doctor report system. 6. When a patient suffers from syphilis and gonorrhea both sexually transmitted diseases, it is needed to fill out a report card and only reported syphilis. 7. When a patient is suffering from syphilis and gonorrhea two sexually transmitted diseases, it is need to fill in two reports card and each STD fill a report card. 8. Syphilis case report by the first doctor is report that the first doctor makes a diagnosis for the patient. 9. The syphilis case report reported by the first doctor is the report that the admissions doctor diagnosis. 10. Referral syphilis cases need to be reported. 11. When the doctor fills the syphilis case "infectious disease report card" accidentally wrong, it cannot modify the card. 12. When the doctor to fill the syphilis case "infectious disease report card" accidentally wrong, the report can be modified and need the doctor should sign. 13. Laboratory test positive cases of syphilis should be reported. if not reported, it sentenced to omission. 14. When the doctor to fill the syphilis case "infectious disease report card" errors, should be modified by the prevention and control staff. 15. Medical institutions security personnel (epidemic management staff) in the verification of syphilis cases "infectious disease report card" found miss cases of classification, Security personnel should be directly on the fill. 16. Medical institutions security personnel (epidemic management staff) in the verification of syphilis cases "infectious disease report card" found miss cases of classification, Security personnel should promptly notify the card doctors, and the doctor needs to make up. 17. Medical institutions doctors are obliged to accept to check by the security personnel (epidemic management personnel) for the syphilis case "infectious disease report card". 18. After Outpatient doctors diagnosed syphilis cases, when the outpatient log was filled the syphilis diagnosis, it is not need to fill the syphilis staging. 19. After Outpatient doctors diagnosed syphilis cases, when the outpatient log was filled the syphilis diagnosis, it is need to fill the syphilis staging. 20. After doctors diagnosed syphilis cases, when it is need to fill the hospital medical records of the syphilis diagnosis, it is need to fill the syphilis staging. 21. Report syphilis cases are divided into early syphilis and late syphilis. 22. Syphilis cases should be reported within 24 hours after diagnosis. 23. Syphilis cases should be reported within 3 days after diagnosis. 24. Syphilis cases that detected positive should be reported within 24 hours. 25. Doctors that do not have the ability to diagnose syphilis suspected syphilis cases should referral or consult other professional doctor. 26. For syphilis referral cases should be reported by the original referral doctor. 27. For syphilis referral cases should be right diagnosis and satisfy requirements of the report 28. Syphilis consultation cases should be reported by the consultation doctor. 29. Syphilis consultation cases are reported by the original admissions doctors and consultation doctors. 30. Syphilis referral cases are reported by the original admissions doctors and consultation doctors. 31. According to the current Ministry of Health issued the diagnostic criteria, syphilis diagnosis cases classified as confirmed cases, clinical diagnosis. 32. According to the current Ministry of Health issued the diagnostic criteria, syphilis diagnosis cases classified as confirmed cases and suspected cases. 33. According to the current Ministry of Health issued the diagnostic criteria, syphilis diagnosis cases classified as clinical diagnosis cases and suspected cases. 34. According to the current Ministry of Health issued diagnostic criteria, syphilis diagnosis cases classified as clinical diagnosis of cases and pathogen carriers. 35. According to the current Ministry of Health issued diagnostic criteria, syphilis diagnosis cases classified as confirmed cases and pathogens carriers. 36. Suspected cases of syphilis reported by the network should be corrected as far as possible by follow-up. 37. Cases of syphilis that cannot be corrected should be removed on the network. 38. Two types of syphilis serum antibody test cases should be positive syphilis report. 39. A hospital test for surgical patients, pregnant women and syphilis screening, its positive results were reported immediately syphilis cases. 40. A hospital inspector found a patient TPPA and RPR test results are positive, and just to see the doctor have a vacation. The inspectors immediately fill out the "infectious disease report card" reported syphilis cases to prevent omission. 41. A hospital health insurance personnel (epidemic management staff) check the reported syphilis case card every working day. One day found that a heart hospital inpatient department doctor reported a case of a syphilis. The security personnel suspected the doctor reported the error and contact the heart physician, asked whether the patient's genital area ulcer performance. Doctors say no clinical manifestations and carry out the "infectious disease four" screening found that TPPA and RPR positive test. It is reported as syphilis. Because the patient does not have any clinical manifestations, the anti-personnel advise the doctor to revise the report and revise the case to conceal the syphilis on the network. 42. A top three hospital dermatologist treatment a case of secondary syphilis cases in September 2012.The patient go to the hospital follow-up review in October 2014. the results found that the patient TPPA and RPR is still positive, RPR titer of 1 ︰ 4. Dermatologist consulted whether the staff of the security department should be reported. Protection staff said that the case span of more than 2 years and need to report the disease, the doctor will report as recessive syphilis. 43. Non-Treponema pallidum serum test RPR titer in 1: 8 following the first diagnosis of syphilis cases do not need to report. 44. A patient was carried out syphilis serum test, the results of TPPA and RPR were positive, asked the history of non-marital sexual behavior history, past no syphilis history, physical examination did not find any syphilis clinical manifestations. The admissions doctor said the case did not carry out syphilis cerebrospinal fluid test. It is not the case diagnosed as hidden syphilis and it does not case reports. 45. A methadone treatment clinic, a drug addicts at the same time to carry out HIV and syphilis serum test, the results of ELISA and TRUST are positive, the staff immediately fill out the infectious disease report card, reported as hidden syphilis confirmed cases. 46. A central blood bank will report all syphilis seropositive blood donors as recessive syphilis. 47. For syphilis sera positive people of inpatients, preoperative patients, maternal and so on, Should consult a professional doctor of dermatology and sexually transmitted diseases, and the consultation doctor need to determine whether carry out case reports. 48. RPR or TRUST titer in the 1: 8 or more syphilis cases need to be reported. 49. Level of medical institutions below the secondary level cannot report syphilis cases. 50. Level of medical institutions above the secondary level have certificate to report syphilis cases. 51. Private medical institutions cannot report syphilis cases. 52. Private medical institutions also have legal obligations to report cases of syphilis according to the Law on Infectious Diseases. 53. Asymptomatic neonatal syphilis should be diagnosed and reported as recessive syphilis. 54. More than 70 years old people diagnosed with syphilis do not need to report. 55. Combined with AIDS syphilis cases, only reported AIDS, do not report syphilis. 56. Mothers who are over 2 years of age are diagnosed with syphilis and should be reported to three syphilis. 57. Some people without syphilis clinical manifestations, past no syphilis diagnosis and the test results for the RPR-positive and TPPA negative, should be reported as recessive syphilis. 58. Blood vessels on the blood donors screening of syphilis serum test positive patients should not report disease, should be referral. 59. A patient was diagnosed with secondary syphilis two years ago. The RPR positive titer was 1: 2, TPPA was positive, and the time span was 2 years. The doctor reported it as recessive syphilis 60. There are currently more than 20 kinds of diseases that can transmitted through sexual pathways. 61. Sexually transmitted diseases in addition to sexual contact spread, and also can spread by the pollution of living utensils. 62. To carry out voluntary prenatal medical examination of the place, should be syphilis counseling and testing services as one of the contents. 63. In the process of childbirth, sexually transmitted diseases can also spread through the birth canal. 64. Eat with syphilis patients, handshake and other daily contact will not be infected with syphilis. 65. Infectious disease patients are more easy infection and transmission of HIV. 66. The body has a lifetime immune to sexually transmitted diseases, once again will not be infected again. 67. Latex condoms must not use lubricants for lubricants. 68. Not all lesions that occur in the genital area are sexually transmitted diseases. 69. The plan of China's prevention and control of syphilis put forward the congenital syphilis report incidence of national should below 35/10 million live births in the end of 2015.   **Questions about clinical treatment of syphilis**  **1. Single choice**   1. How many years was division from early and late syphilis in China.   A. 1 year B. 2 years  C. 3 years D. 4 years   1. Early syphilis not include:   A. First syphilis B. second syphilis  C. Early latent syphilis D. third syphilis   1. About syphilis, which of the following is wrong:   A. Syphilis is a chronic systemic of sexually transmitted disease  B. Late syphilis is more contagious  C. Submerged syphilis patients without syphilis-related symptoms  D. Late syphilis is more destructive to the tissue   1. About regard to the biological characteristics of Treponema pallidum, which of the following is wrong:   A. It is a small and slender spiral microbes  B. Refractive stronger than other spirochetes  C. Can be a regular exercise  D. Gram stains are easily observed with an optical microscope   1. About the biological characteristics of Treponema pallidum, which of the following is wrong:   A. Treponema pallidum is a common animal and people  B. Treponema pallidum cannot be cultured out body  C. The most suitable survival temperature is 37 ℃  D. General disinfectants are easy to kill Treponema pallidum   1. About the incidence of syphilis, which of the following is wrong:   A. it has relationship between The incidence of syphilis and Treponema pallidum in the human body a lot of breeding  B. Sexual contact with Treponema pallidum through the damaged skin and mucous membranes to pass sexual partners  C. Early syphilis can produce anticardiolipin antibodies and anti-Treponema pallidum antibodies  D. Anti-Treponema pallidum antibody has an immunoprotective effect on the body   1. The incubation period for primary syphilis is:   A 3～5 days B 2～4 weeks  C 1～2 months D 2～3 months   1. The main clinical manifestations of primary syphilis are:   A. Genital ulcers B. Urethral secretions  C. Genital multiple blisters D. Genital neoplasm   1. The typical manifestation of chancre is the following characteristics, except:   A. Single ulcer B. Ulcer surface mucus purulent discharge  C. Palpation with cartilage hardness D. No conscious pain and tenderness   1. Hard chancre subsided time is usually:   A 1～3 weeks B 3～8 weeks  C 8～12 weeks D 12～16 weeks   1. About the characteristics of chancre, which of the following is wrong:   A. Occurrence of Treponema pallidum inoculation site  B. Male and male sexes are common in the perianal and rectum  C. Typical hard chancre is a single ulcer  D. Atypical chancre is painless and tender   1. About the characteristics of a syphilis near the lymph nodes, which of the following is wrong:   A. In the chancre occurred after 1 to 2 weeks  B. Surface skin without swelling and heat pain  C. The lymph nodes contain Treponema pallidum  D. Swollen lymph nodes are soft and can occur   1. which is Chancre of clinical manifestations of syphilis:   A. Phase I syphilis syphilis B. Phase II syphilis syphilis  C. Phase III syphilis syphilis D. Fetal syphilis   1. Phase II syphilis generally occurs after Treponema pallidum infection:   A 1～3 weeks B 3～7 weeks  C 7～10 weeks D 10～14 weeks   1. Which is the Flat condyloma of the clinical manifestation:   A. Phase I syphilis syphilis B. Phase II syphilis syphilis  C. Phase III syphilis syphilis D. Nerve syphilis   1. About the second stage of syphilis skin and mucous membrane damage, which of the following is wrong:   A. Rash diversity, widely distributed and symmetrical  B. No symptoms or symptoms minor  C. Destructive to the organization  D. Highly contagious   1. About the second stage of syphilis skin and mucous membrane damage, which of the following is wrong:   A. The most common pimples are seen  B. Palmoplantar skin lesions are dark red or light brown circular desquamation rash  C. Skin shows "raw ham" color D. Mostly distributed in the trunk and limbs   1. Flat warts are characterized by the following characteristics, except:   A. Occur in the perianal, external genital area B. The lesion is pimples or plaque  C. Clear boundaries D. without Treponema pallidum   1. The following is the clinical manifestations of secondary syphilis, except:   A. Superficial lymph nodes B. Eye syphilis  C. Pleomorphic skin and mucous membrane damage D. Tree swollen   1. The following is a description of the clinical manifestations of secondary syphilis, except:   A. Accompanied by headache, nausea and other precursor symptoms  B. Can occur moth-like hair loss  C. The occurrence of neurosyphilis is mostly asymptomatic  D. Bone and joint pain increases during daytime and activity   1. What is clinical manifestations of syphilis:   A. Phase I syphilis syphilis B. Phase II syphilis syphilis  C. Phase III syphilis syphilis D. Nerve syphilis   1. Phase III skin and mucous membrane syphilis is characterized by:   A. more lesions, distribution symmetry  B. Rapid progress, not self-healing  C. It is not easy to ulcers  D. Inflammation and systemic symptoms are mild   1. Paralytic dementia is the following type of clinical manifestations of syphilis:   A. Phase I syphilis syphilis B. Phase II syphilis syphilis  C. Phase III syphilis syphilis D. Nerve syphilis   1. Neurosyphilis can be divided into the following types, except:   A. Meningeal nerve syphilis  B. Meningeal blood vessels syphilis  C. Brain parenchymal syphilis  D. Eye syphilis   1. What is the clinical manifestation of spinal tuberculosis:   A. Phase I syphilis syphilis B. Phase II syphilis syphilis  C. Phase III syphilis syphilis D. Nerve syphilis   1. Paralytic dementia has the following clinical manifestations, with the exception of:   A Occurred infection after 10 to 20 years  B Mental retardation, inattention  C Arrow pupil D Optic nerve atrophy   1. Spinal tuberculosis has the following clinical manifestations, except:   A. Lightning pain B. Optic nerve atrophy  C. feeling abnormal D. Tactile sensation and temperature disorder   1. About latent syphilis, which of the following is wrong:   A. Without any clinical symptoms B.Syphilis seropositivity was positive  C. Infection period within 2 years for early latent syphilis  D. Stable condition, no infectious   1. The following is the clinical manifestations of early congenital syphilis, except:   A. Nutritional disorders B. Hard chancre  C. Blister or blister rash D. Flat condyloma   1. About congenital syphilis, which of the following is true:   A. Pregnant women infected longer, the more likely to spread the fetus  B. Can occur chancre  C. Rhinitis is the most common early symptoms  D. Not easy to involve the nervous system   1. Syphilis serum fixation means:   A. After the treatment of syphilis, spirochete antibody test in a certain period of time does not turn negative  B. After the treatment of syphilis, non-spirochete antibody test in a certain period of time does not turn negative  C. Untreated syphilis patients, 3 consecutive non-spirochete antibody titers maintained at the same level  D. Untreated syphilis patients, 3 consecutive times to detect spirochete antibody titers remain at the same level   1. The following is the purpose of syphilis treatment, except:   A Each period of syphilis are required to kill the body Treponema pallidum, eliminate or reduce the infectivity  B Early syphilis damage disappeared, to achieve clinical cure  C Each period of syphilis are required serum negative  D Pregnancy syphilis to make the fetus from infection   1. Early treatment of syphilis is:   A. Benzathine penicillin G 1.2 million U, on both sides of the buttocks intramuscularly, once a week, a total of 2 times  B.Benzathine penicillin G 2.4 million U, on both sides of the buttocks intramuscularly, once a week, a total of 2 times  C. Benzathine penicillin G 4.8 million U, on both sides of the buttocks intramuscularly, once a week, a total of 2 times  D. Benzathine penicillin G 1.2 million U, on both sides of the buttocks intramuscularly, once a week, a total of 5 to 6 times   1. Early syphilis preferred treatment for:   A. Penicillin sodium B. Penicillin potassium  C. Ceftriaxone D. Benzathine penicillin   1. Early syphilis penicillin allergy treatment program is:   A Doxycycline 100mg, 2 times a day, oral, treatment 15d  B Azithromycin 500mg, day 1, oral, treatment 15d  C Erythromycin 500mg, 4 times a day, oral, treatment 15d  D Doxycycline 200mg, 2 times a day, oral, treatment 15d   1. Advanced syphilis recommended treatment plan is:   A. Benzathine penicillin G 1.2 million U, on both sides of the buttocks intramuscularly, once a week, a total of 3 times  B. Benzathine penicillin G 1.2 million U, on both sides of the buttocks intramuscularly, once a week, a total of 6 times  C. Benzathine penicillin G 4.8 million U, on both sides of the buttocks intramuscularly, once a week, a total of 3 times  D. Benzathine penicillin G 240 million U, on both sides of the buttocks intramuscularly, once a week, a total of 3 times   1. Advanced syphilis on penicillin allergy treatment program is:   A Doxycycline 100mg, 2 times a day, oral, treatment 30d  B Azithromycin 250mg, 2 times a day, oral, treatment 30d  C Azithromycin 500mg, day 1, oral, treatment 30d  D Erythromycin 500mg, 4 times a day, oral, treatment 30d   1. Neurosyphilis with water penicillin treatment, the dose range:   A 4.80～6.4 million U/day B 6.4～8 million U/day  C 10～12 million U/day D 18～24 million U/day   1. The following description of the treatment of early syphilis is appropriate:   A. Benzazole penicillin intravenously  B. Benzathine penicillin intramuscular injection  C. Intravenous infusion of penicillin sodium  D. Penicillin sodium intramuscular injection   1. It has been confirmed that Treponema pallidum has been generally resistant to the following drugs:   A.Benzathine penicillin B. Procaine penicillin  C. Macrolides D. Tetracycline   1. In accordance with the drug metabolic half-life arrangement, which of the following is correct:   A. Water penicillin> procaine penicillin> benzathine penicillin  B. Benzathine penicillin> procaine penicillin> water agent penicillin  C. Water penicillin> benzathine penicillin> procaine penicillin  D. Benzathine penicillin> water agent penicillin> procaine penicillin   1. About ceftriaxone treatment of early syphilis, which of the following statements is wrong:   A. Half-life is long, so it can be administered once a week  B. The penetration of cerebrospinal fluid is better  C. Only as an alternative treatment  D. Animal model studies have a cytotoxic effect on Treponema pallidum   1. Which of the following statements is wrong about the Kyrgyzstan reaction:   A. Often occurs in the first dose of anti-syphilis after treatment for several hours  B. There is fever, cold, general malaise, headache, musculoskeletal pain and so on  C. Pregnant women can cause premature birth or fetal intrauterine asphyxia  D. The incidence of late syphilis is higher than that of early syphilis   1. If syphilis treatment is effective, non-spiral test negative time is generally:   A. Phase I syphilis 3 months; Phase II syphilis 6 months  B. Phase I syphilis 6 months; Phase II syphilis 12 months  C. Phase I syphilis 9 months; Phase II syphilis 18 months  D. Phase I syphilis 12 months; Phase II syphilis 24 months   1. Early syphilis treatment effective judging criteria, in 3 to 6 months when the non-spiral test titer should be at least several times lower than before treatment:   A 2 B 4  C 8 D 12   1. How long should follow-up of early syphilis treatment:   A 6 months B 6 months～12 months  C 12～24 months D 24～36 months   1. About the treatment of pregnant women, syphilis Which of the following is wrong:   A treatment  B After treatment in pregnancy should be checked once a month non-spiral test titer  C Penicillin allergy with erythromycin or doxycycline treatment  D Erythromycin can not pass through the placenta, the fetal treatment is invalid   1. Adult prophylactic anti-syphilis treatment program recommended as:   A Benzathine penicillin G 1.2 million U, intramuscularly, once a week, a total of 2 times  B Benzathine penicillin G 240 million U, intramuscularly, once a week, a total of 2 times  C Benzathine penicillin G 1.2 million U, 1 intramuscular injection  D Benzathine penicillin G 240 million U, 1 intramuscular injection   1. Early congenital syphilis cerebrospinal fluid abnormalities of the treatment program is:   A Water infusion of penicillin G, 10 million U ~ 15 million U / (kg • d), within 7 days after birth of newborns, each 50,000 U / kg, intravenous injection every 12 hours 1; Infants every 8 hours 1, until the total course of treatment 10 ~ 14 d  B Enamel penicillin, 100,000 U ~ 150,000 U / (kg • d), newborns within 7 days after birth, with a daily dose of 50,000 U / kg, once every 12 hours; Every 8 hours 1, until the total course of 10 ~ 14 d  C Camphor penicillin, 100,000 U ~ 150,000 U / (kg • d), newborns within 7 days after birth, every 50,000 U / kg, intravenous every 12 hours 1; Of the baby every 8 hours 1, until the total course of 10 ~ 14 d.  D Erythromycin, 100,000 U ~ 150,000 U / (kg • d), newborns within 7 days after birth, every 5 000 U / kg, intravenous every 12 hours 1; born 7 days after the baby Every 8 hours 1, until the total course of 10 ~ 14 d   1. Neurosyphilis changes in cerebrospinal fluid, which of the following is wrong:   A White blood cell count ≥ 5 × 10^6^ / L  B Protein> 500mg / L  C In the absence of conditions to do VDRL circumstances, can be replaced with RPR  D The specificity of the spirochetes test is stronger   1. In the prenatal examination, the best period for syphilis testing for pregnant women is:   A Early pregnancy B Mid - pregnancy  C Late pregnancy D Time of birth   1. The following description of pregnancy syphilis is wrong:   A The main route of maternal infection with syphilis is sexually transmitted  B Pregnancy syphilis often have obvious clinical symptoms  C Pregnancy syphilis easily lead to abortion, stillbirth, congenital syphilis or neonatal death and other adverse outcomes  D At any stage of pregnancy, Treponema pallidum can infect the fetus   1. Which of the following test results can determine whether pregnant women are currently infected with syphilis and are contagious:   A RPR（－） TPPA（－） B RPR（＋） TPPA（－）  C RPR（＋） TPPA（＋） D RPR（－） TPPA（＋）   1. The following description of congenital syphilis is correct:   A. Syphilis infection mothers born to children with RPR positive, can be diagnosed as congenital syphilis  B. Syphilis infected mothers born at 12 months of age TPPA positive, can be diagnosed as congenital syphilis  C. RPR titer low level (such as 1: 1), followed up to 18 months of age, TPPA remained positive, can be diagnosed as congenital syphilis  D.Dark field microscope did not detect Treponema pallidum, can rule out congenital syphilis   1. What cases of syphilis infection in pregnant women that born to children can not be diagnosed as congenital syphilis?   A Dark field microscopy detected Treponema pallidum  B TPPA test positive, RPR titer higher than the mother before delivery 4 times the titer  C Treponema pallidum IgM antibody test positive  D TPPA test positive, RPR test negative   1. The following description of the preventive treatment of children born to syphilis in pregnant women is wrong:   A Mothers do not receive full treatment during pregnancy, adequate treatment, children should receive preventive treatment  B Mothers are treated with non-penicillin, and children should receive prophylactic treatment  C After the application of benzathine penicillin G, 50,000 units / kg body weight, sub-double gluteal muscle injection  D Prophylactic treatment should last for 15 days  2. **Judgment questions**   1. Early syphilis is the Syphilis of the infected treponema within 3 years. 2. Early syphilis includes primary syphilis, secondary syphilis and latent syphilis. 3. Late syphilis includes late benign syphilis, late cardiovascular syphilis and late syphilis latent. 4. Neurosyphilis can occur in early and late syphilis. 5. The incubation period of the chncre syphilitique takes between 4-8 weeks. 6. Typical hard chancre has no obvious pain or tenderness. 7. The chancre only occurs in the genitals or the anus. 8. An enlarged lymph node in the stage of the first stage of syphilis is painful, and the surface is red and swollen. 9. In the first stage of syphilis, if the infection is less than 2-3 weeks, the non-spiral-body test can be negative. 10. Skin lesions of secondary syphilis can simulate any skin lesions. 11. Condyloma latum is a characteristic damage of secondary syphilis. 12. Oral mucosais a characteristic damage of tertiary syphilis. 13. Alopecia vermiculata is a characteristic damage of secondary syphilis. 14. Skin lesions of secondary syphilis are widely, symmetrical and not special. 15. Superficial lymph nodes can be enlarged in the secondary syphilis. 16. Neurosyphilis can occur in the secondary syphilis. 17. It is easy to find treponema pallidum in the Condyloma lata or [moist papule](http://www.youdao.com/w/moist papule/" \l "keyfrom=E2Ctranslation) of secondary syphilis. 18. Non-spiraling serological test was 100% positive in the second stage of syphilis. 19. Gumme is a characteristic damage of secondary syphilis. 20. The upper palate and septum perforation and saddle nose are usually seen in tertiary syphilis. 21. Meningoencephalitis is a type of neurosyphilis. 22. Cerebral parenchymal syphilis includes paralytic dementia and the tuberculosis of the spinal cord. 23. The characteristic of tuberculosis of the spinal cord is the concentration and personality disorder. 24. Abnormal white blood cell count decreased in cerebrospinal fluid during neurosyphilis. 25. The non-treponemal tests of Cerebro-spinal fluid show high susceptibility in the diagnosis of neurosyphilis. 26. The treponemal tests of Cerebro-spinal fluid show high specificity in the diagnosis of neurosyphilis. 27. The clinical feature of early congenital syphilis is similar to that of adult with secondary syphilis. 28. The clinical manifestations of late congenital syphilis are similar to that of adults with tertiary syphilis. 29. Congenital syphilis can be diagnosed by the positive test of neonatal non-treponemal serology. 30. Congenital syphilis can be diagnosed by the positive test of Neonatal treponema pallidum IgM antibody. 31. If the antibody titer of neonatal non-helix is higher than that of the mother, then the newborn can be diagnosed with congenital syphilis. 32. Congenital syphilis can be diagnosed by the positive test of neonatal treponemal serology. 33. If a newborn followed up to 15 months is test for a treponemal serolog positive, then the newborn can be retrospectively diagnosed with congenital syphilis. 34. The earlier treatment the patient with syphilis received, the better treatment effect. 35. The irregular treatment of syphilis can increase recurrence and lead to advanced damage earlier. 36. Treatment of early syphilis patients needs to be tracked for a year. 37. Benzathine penicillin is the best choice for the treatment of patient with early syphilis. 38. Benzathine penicillin is the best choice for the treatment of patient with [neurosyphilis](http://www.youdao.com/w/neurosyphilis/#keyfrom=E2Ctranslation). 39. Aqueous penicillin is the best choice for the treatment of patient with late syphilis. 40. Benzathine penicillin is the best choice for the treatment of patient with early congenital syphilis and cerebrospinal fluid abnormality. 41. Many treponema spirals have been found to be resistant to penicillin. 42. Many syphilis spirals have been found to be resistant to large ring lactone. 43. The jihai reaction is the persisting positive of the non-treponemal serological test after the treatment of syphilis. 44. Most of the first stage syphilis becomes negative in six months of non-treponemal serological test. 45. The recurrence of serum is that the titer result of non-treponemal test changes from negative to positive or more than four times higher than the previous one, after the treatment of early syphilis. 46. Treatment of patients with cardiovascular syphilis or neurosyphilis needs to be tracked for 3 year or longer. 47. Serum fixation after the anti-plum treatment, non-spirochete antibody titer for a long time without negative. 48. When pregnant women receive maternal health care services for the first time, they should provide free syphilis testing services. 49. Pregnancy syphilis is the standard treatment for pregnant women to provide two courses of treatment, between the two courses of treatment must more than two weeks, The last course of treatment in the third trimester, the use of penicillins, ceftriaxone or erythromycin for treatment. 50. If syphilis infected maternal use of drugs during pregnancy, the children born to their children cannot be preventive treatment. 51. For children born cannot diagnose congenital syphilis,he should be regularly detected and followed up, and timely diagnosis or elimination of congenital syphilis. 52. Pregnant women that infected syphilis in the labor also given a course of treatment. 53. Syphilis infection maternal born children born after Treponema pallidum IgM antibody test was negative, you can rule out congenital syphilis. 54. For cerebrospinal fluid normal congenital syphilis children, can be used benzathine penicillin, according to 50,000 U / kg body weight for a muscle injection.   **Syphilis Testing Questions**  **1. Single choice**   1. Which is the suitable staining method used in the examination of treponema pallidum?   A. Gram stain B. Methylene blue staining  C. Silver stain D. Hematoxylin-eosin staining   1. Which is the suitable method used in the test of nontreponemal antibodies?   A. TPPA B. ELISA  C. RPR D. FTA-ABS   1. What the following thing can be only detected by TPPA test?   A. IgA B. IgG  C. IgG,IgM, IgA, et al D. IgM   1. Generally, what the following specimen doesn’t used in the examination of treponema pallidum?   A. Tissue fluid of skin ulcer B. Amniotic fluid  C. Blood D. Cerebrospinal fluid   1. What the following specimen doesn’t be used in the RPR test?   A. [Serum](http://www.youdao.com/w/serum/#keyfrom=E2Ctranslation) B. Blood plasma  C. [Whole blood](http://www.youdao.com/w/whole blood/" \l "keyfrom=E2Ctranslation) D. Cerebrospinal fluid   1. What the following specimen doesn’t be used in the ELISA test?   A. [Serum](http://www.youdao.com/w/serum/#keyfrom=E2Ctranslation) B. Blood plasma  C. [Whole blood](http://www.youdao.com/w/whole blood/" \l "keyfrom=E2Ctranslation) D. Cerebrospinal fluid   1. What the following method doesn’t belong to etiology detection of treponema pallidum?   A. [Dark-field microscopy](http://www.youdao.com/w/dark-field microscopy/" \l "keyfrom=E2Ctranslation) B. Silver stain  C. Medium culture method  D. Nucleic acid test   1. What is the rotational speed of the horizontal rotator used in PRP or TRUST test?   A. (100±2) rotations per minute B.(100±10) rotations per minute  C. (120±2)rotations per minute  D. (120±10)rotations per minute   1. What is the rotation diameter of the horizontal rotary instrument used in PRP or TRUST test?   A. (25±2)mm B.(18±2) mm  C. (20±2)mm  D. (30±2)mm   1. What is the rotation reaction time of the horizontal rotator in PRP or TRUST test?   A. 5 minute B.8 minute  C. 10 minute   D.12 minute   1. What is the concentration of antigen we need add it in each reaction in PRP or TRUST test?   A. 50 μl B.30 μl  C. 25 μl   D.17 μl   1. What is the following method that often leads to frontal zone phenomenon?   A. ELISA B. RPR  C. TPPA D. FTA-ABS   1. What is the concentration of antigen we need add it in each reaction in PRP test?   A. 50 μl B.30 μl  C. 25 μl   D.17 μl   1. Which isn’t the suitable method used in the test of nontreponemal antibodies?   A. RPR B. ELISA  C. VDRL D. TRUST   1. What is the [dilution ratio](http://www.youdao.com/w/dilution ratio/" \l "keyfrom=E2Ctranslation) of result interpretation hole serum in TPPA test?   A. 1:20 B. 1:40  C. 1:80 D. 1:160   1. What is the antigen used in ELISA test?   A. Recombinant antigen B. Complete treponema pallidum  C. Ultrasonic pyrolysis of treponema pallidum D. lipid content   1. What is the antigen used in TPPA test?   A. Recombinant antigen B. Complete treponema pallidum  C. Ultrasonic pyrolysis of treponema pallidum D. lipid content   1. What is the antigen used in RPR test?   A. Recombinant antigen B. Complete treponema pallidum  C. Ultrasonic pyrolysis of treponema pallidum D. lipid content   1. What is biosafety level (BL) of syphilis serological testing laboratory?   A. BL-1 B. BL-2 C. BL-3 D. BL-4   1. What is the main aim of quality of room control?   A. The effectiveness of detecting approach  B. The accuracy of detecting result  C. The veracity of detecting result  D. The feasibility of detecting approach   1. What is the main aim of internal quality control?   A. The effectiveness of detecting approach  B. The accuracy of detecting result  C. The veracity of detecting result  D. The feasibility of detecting approach     1. What the following thing can be only detected by RPR test?   A. IgA B. IgG  C. IgG,IgM, IgA, et al D. IgM   1. Which is the suitable method used in the test of treponemal antibodies?   A. TPPA B. ELISA  C. RPR D. VDRL   1. Which is not suitable method used in the test of syphilis blood screening?   A. TRUST B. ELISA C. PCR D. TPPA   1. Which is not suitable thing detected by etiology test?   A. Primary syphilis B. Secondary syphilis  C. latent syphilis D. Neurosyphilis   1. What type of syphilis is recommend to be diagnosed by IgM antibody detection of syphilis   A. Congenital syphilis B. Secondary syphilis  C. latent syphilis D. Pregnant syphilis   1. What is the following disease that positive [cerebrospinal fluid](http://www.youdao.com/w/cerebrospinal fluid (CSF)/" \l "keyfrom=E2Ctranslation) VDRL test can support the diagnosis?   A. Primary syphilis B. Secondary syphilis  C. latent syphilis D. Neurosyphilis   1. Which is the following approach that can provide magnitude value for neurosyphilis?   A. Blood TRUST B. Blood ELISA  C. Blood RPR D. [Cerebrospinal fluid](http://www.youdao.com/w/cerebrospinal fluid (CSF)/" \l "keyfrom=E2Ctranslation) VDRL   1. Which is the following approach that need microscope to read the result of the condensate?   A. TRUST B. ELISA  C. RPR D. VDRL   1. Which is the approach for the detection of syphilis serology using whole blood sample?   A. TRUST B. ELISA  C. RPR D. Immunochromatography rapid detection   1. After the infection of treponema pallidum, what is the first detectable antibody method?   A. TRUST B. TPPA  C. RPR D. VDRL   1. Which is the antibody that cannot crosses through the placenta to the fetus in syphilis-positive pregnant women?   A. IgM B. IgG  C. All of the antibodies D. IgA   1. What is the main observation of syphilis dim view microscope?   A. The spirals of characteristic movement  B. Special dye spirals  C. Characteristic inflammatory cells  D. The characteristic pathology changes   1. What is the main content of treponema pallidum that examination of silver-plated staining was used to observe?   A. The spirals of characteristic movement  B. Special dye spirals  C. Characteristic inflammatory cells  D. The characteristic pathology changes   1. What is the positive rate of non-specific treponema pallidum in secondary syphilis?   A. <90% B. 100%  C. <95% D. >95%   1. What is the positive rate of treponema pallidum in secondary syphilis?   A. <90% B. 100%  C. <95% D. >95%   1. Which is the following quantitative test method that is rarely referred to syphilis therapeutic effect judgment?   A. TRUST B. TPPA  C. RPR D. VDRL   1. What is the maximum follow-up time for treponemal antibodies that are value for diagnosis of congenital syphilis?   A. Six month B. Nine month  C. Twelve month D. Eighteen month   1. If frontal zone phenomenon is suspected in RPR or TRUST test, how many times the serum should be diluted at least?   A. 1:2 B. 1:4  C. 1:8 D. 1:16   1. What instrument should be adopted to mix antigen and antibody in TPPA test?   A. Horizontal gyro B. Hand blender  C. 96-well-plate oscillator D. suspended implement   1. What instrument should be adopted to mix antigen and antibody in RPR or TRUST test?   A. Horizontal gyro B. Hand blender  C. 96-well-plate oscillator D. suspended implement   1. What dose biologic false positive syphilis serology means in sample in RPR/TRUST test?   A. There are non-specific antibodies of syphilis  B. There aren’t non-specific antibodies of syphilis  C. There are specific antibodies of syphilis  D. There are non-specific antibodies and specific antibodies of syphilis   1. When the same sample was detected by using both [quantitative RPR](http://www.youdao.com/w/quantitative determination/" \l "keyfrom=E2Ctranslation) and quantitative TRUST, what the following opinion about the titer is right ?   A. They do not necessarily correspond  B. They do necessarily correspond  C. The titer of RPR must be higher than TRUST  D. The titer of TRUST must be higher than RPR   1. So far, what is the gold standard method for antibody test of specific treponema pallidum?   A. ELISA B.TPPA  C. Chemiluminescent immunoassay D. Immunochromatography rapid detection   1. What kind of specimen is not recommended for the detection of treponema pallidum by PCR test?   A. [Interstitial fluid](http://www.youdao.com/w/interstitial fluid/" \l "keyfrom=E2Ctranslation) B. Lymph fluid  C. Cerebrospinal fluid D. Blood   1. Generally, what kind of method is recommended to use in large sample syphilis screening?   A. ELISA B.TPPA  C. RPR/ TRUST D. Immunochromatography rapid detection   1. When RPR test is positive in syphilis screening, what kind of test can be used to exclude biologically false positivity?   A. TRUST B.TPPA  C. VDRL D. RPR   1. What is the kind of antibodies that cannot pass through the blood-brain barrier?   A. IgM B. IgG  C. All of the antibodies D. IgA   1. What kind of syphilis can be clinical diagnosed through IgM antibody detection of syphilis?   A. Primary syphilis B. Secondary syphilis  C. latent syphilis D. Neurosyphilis   1. According to the degree of pathogenicity, the pathogenic microorganism of treponema pallidum was classified as?   A. First kind B. Second kind  C. Third kind D. The fourth kind  **2. Judgment questions**   1. In addition to some of the early syphilis, syphilis spirochete specific antibodies are often lifelong positive in general syphilis patients whether or not have received effective anti-plum treatment. 2. RPR or TRUST can only used for the screening of syphilis serology. 3. In the diagnosis of secondary syphilis, both TPPA and RPR test were 100% positive. 4. In the diagnosis of recessive syphilis, RPR titer should be above 1:8. 5. In the laboratory examination of congenital syphilis, cord blood can be collected for syphilis serological test. 6. The blood samples of the subjects were negative, indicating that the patient didn’t infected with treponema pallidum. 7. After following up baby treated for syphilis for 18 month, baby had infected with treponema pallidum if TPPA test was positive and RPR was negative. 8. Manual shaking can be used in PRP or TRUST quantitative test. 9. If TPPA test was negative and RPR was positive, the patient can be excluded from the treponema pallidum. 10. Cerebrospinal fluid VDRL test results must be positive in patient with Neurosyphilis. 11. If the concentration is not obvious in RPR test, the reading of results can be extended to 10 minutes. 12. If the sample was tested negative for RPR or TRUST, there is no need to dilution serum for retest. 13. If dark field examination revealed no treponema pallidum in specimen of a patient's genitals, the patient can be excluded from the treponema pallidum. 14. Frontal zone phenomenon occurred in the detection of the syphilis antibody, indicating that there is necessary to dilution serum for TPPA test. 15. Both TPPA and RPR were positive in neonatal sera, indicating that the newborn has been infected with treponema pallidum. 16. As there was a window period for syphilis serological test, Blood samples were detected by PCR tested in order to improve the sensitivity of early syphilis detection. 17. The rotation speed of the horizontal rotary instrument used in the RPR/TRUST test should be (120±2) rotation per minute. 18. The pathogen was tested positive, which can be used as a basis for diagnosis of syphilis. 19. Any kind of serological detection method can be used to test for syphilis screening. 20. TPPA is the method of detecting syphilis serological test, so people can be diagnosed as syphilis patient if TPPA was positive. 21. The detection of IgM of syphilis is of great significance to the diagnosis of congenital syphilis. 22. Because RPR and TRUST have the same principle, so the results of quantitative experiment of two methods must be consistent. 23. TPPA test is positive and RPR test was negative maybe means that nonspecific antibodies are in the window, for that patient needed to be follow up to exclude early syphilis. 24. ELISA and other specific treponema pallidum antibody tests can be used to detect the patients who were infected with syphilis. 25. Sero-resistance means that TPPA test keep positive in patients after the standardized treatment of syphilis. 26. The purpose of laboratory quality control is to improve the precision of laboratory test results. 27. TPPA titer detection is an important index for clinical treatment of syphilis. 28. If TPPA test is positive and RPR test was negative, it usually means that RPR test was false negative. 29. IgM antibody detection can replace the conventional TPPA and other treponema pallidum test. 30. As long as the test for syphilis is operated according to the instructions of the kit, test standard operating procedure (SOP) is not required. 31. Treponema pallidum ELISA test were positive, indicating that here must be a specific antibody of treponema pallidum in the specimen. 32. The clinical etiology of syphilis was mainly use of histological fluid specimens of the secondary syphilis skin lesions. 33. All blood samples should not be used in TPPA or ELISA test. 34. When the test results of RPR or TRUST are suspicious, the agglutination results can be observed under the microscope. 35. We cannot configure the internal quality control specimens of syphilis serological test by ourselves in the laboratory. 36. If the RPR/TRUST horizontal rotary instrument has been set at a speed, normally we don't have to check periodically. 37. Using regular syphilis serological test such as TPPA or RPR, IgG antibodies can only be detected. 38. There is no significance for the diagnosis of neurosyphilis by IgM antibody detection. 39. The test of syphilis serology should be performed in the first class biosafety laboratory. 40. All blood samples should not be used in immunochromatography rapid detection. 41. Silver plating should not be used in detection of treponema pallidum. 42. Because RPR and TRUST have the same principle, so we can develop the same standard operating procedure(SOP) for both two tests in the laboratory. 43. The results of laboratory quality control are all qualified every year, indicating the precision of laboratory test results are well. 44. Gelatin particles and [red blood corpuscle](http://www.youdao.com/w/red blood corpuscle/" \l "keyfrom=E2Ctranslation) are used as antigen sensitized vector in TPPA test and TPHA test respectively, so the [stability](http://www.youdao.com/w/stability/#keyfrom=E2Ctranslation) of TPPA test is better than TPHA test. 45. All syphilis serological tests are not allowed to be read outside of the prescribed time. 46. In the report of the testing result, we only need to point that whether the specific antibody or non-specific antibody was detected without specific method. 47. When conducting the TPPA test, the specimen and antigen should be mixed by the oscillator. 48. The frontal zone phenomenon of RPR or TRUST test can be eliminated by repeated qualitative test. 49. When early syphilis patients were tested for syphilis, general specific antibodies was tested positive prior to non-specific antibodies. 50. The diagnosis of congenital syphilis can be made by using specific antibody detection for 18 months. |
| --- |
