# Supplementary material for: The current status of syphilis prevention and control in Jiangsu province, China: A cross-sectional study
Source: PLoS One. 2017 Aug 24;12(8):e0183409. doi: 10.1371/journal.pone.0183409 (PMC5570431; doi:10.1371/journal.pone.0183409)
Supplement: S2 Table — (DOC) [file pone.0183409.s002.doc]

**S2 Table. Questionnaire about Syphilis Prevention Knowledge**

| Address:  **People type: ①city people ②country-side people ③rural-to-urban migrants ④FSW ⑤MSM**  Number of questionnaire：口口口口 GB code：口口口口口口  ──────────────────────────────────  Dear, an investigation that people's awareness of some health problems is going to improve our work. This investigation does not need to sign, and we will keep your answer confidential. I hope your answer is real knowledge, and I can provide health advice for you after the investigation. Thank you!  **Syphilis prevention knowledge problem:**   1. Is syphilis spread through sexual contact primarily?   ①Yes ②No ③Do not know   1. Can syphilis be cured?   ①Yes ②No ③Do not know   1. A person who looks healthy will be syphilis?   ①Yes ②No ③Do not know   1. If proper use of condoms, it can prevent the spread of syphilis?   ①Yes ②No ③Do not know   1. Does syphilis increase the spread of AIDS?   ①Yes ②No ③Do not know   1. Syphilis patients need to go to the hospital check?   ①Yes ②No ③Do not know   1. Will pregnant women that infected with syphilis be transmitted to the fetus?   ①Yes ②No ③Do not know   1. Will eating with syphilis, handshake and other daily contact spread syphilis?   ①Yes ②No ③Do not know  Investigator sign： Leader sign：  Supervisor sign： Time： |
| --- |
