# Supplementary material for: The current status of syphilis prevention and control in Jiangsu province, China: A cross-sectional study
Source: PLoS One. 2017 Aug 24;12(8):e0183409. doi: 10.1371/journal.pone.0183409 (PMC5570431; doi:10.1371/journal.pone.0183409)
Supplement: S3 Table — (DOC) [file pone.0183409.s003.doc]

**S3 Table. Survey on provider-initiated syphilis testing and counseling (PISTC) services in STD clinics**

Area name: Province City County

Unit who collected information:

**Collection time:**

| Total number of STD clinics in jurisdictions | Among them: the number of STD clinics that carried out the detection of syphilis | | Proportion (%) | Remark |
| --- | --- | --- | --- | --- |
|  |  | |  |  |
| The name of medical institution | The number of outpatients who were tested for syphilis in the department of dermatology in the fourth quarter of 2015 | The number of patients with STD in the fourth quarter of 2015 | | the percentage of provider-initiated syphilis testing and counseling services |
|  |  | The number of registered patients with STD | The number of outpatient in the department of dermatology ×3% |  |
|  |  |  |  |  |
|  |  |  |  |  |
|  |  |  |  |  |
|  |  |  |  |  |
|  |  |  |  |  |
| Total |  |  |  |  |
| Other explanations: the number of patients with STD in the fourth quarter of 2015 is the number of registered patients with STD or three percent of the number outpatient in the department of dermatology. | | | | |
