# Supplementary material for: The current status of syphilis prevention and control in Jiangsu province, China: A cross-sectional study
Source: PLoS One. 2017 Aug 24;12(8):e0183409. doi: 10.1371/journal.pone.0183409 (PMC5570431; doi:10.1371/journal.pone.0183409)
Supplement: S4 Table — (DOC) [file pone.0183409.s004.doc]

**S4 Table. Survey on the ratio of syphilis-positive patients who had received standard treatment**

Area name: Province City County

Unit who collected information:

**Collection time:**

| The name of medical institution | Prescriptions investigated | Long-acting penicillin prescriptions ÷0.75 | Standard treatment ratio(%) |
| --- | --- | --- | --- |
| 1. |  |  |  |
| 2. |  |  |  |
| 3. |  |  |  |
| …. |  |  |  |
|  |  |  |  |
|  |  |  |  |
|  |  |  |  |
|  |  |  |  |
| Total |  |  |  |
